# Dying during the COVID-19 Pandemic in Sweden: Relatives’ Experiences of End-of-Life Care (the CO-LIVE Study)

**DOI:** 10.3390/ijerph192316146

**Published:** 2022-12-02

**Authors:** Christel Hedman, Carl Johan Fürst, Birgit H. Rasmussen, Agnes van der Heide, Maria E. C. Schelin

**Affiliations:** 1 Department of Molecular Medicine and Surgery, Karolinska Institutet, Anna Steckséns gata 53, SE-17176 Stockholm, Sweden; 2 R&D Department, Stockholms Sjukhem Foundation, Mariebergsgatan 22, SE-11219 Stockholm, Sweden; 3 Department of Clinical Sciences Lund, Lund University, BMC, Sölvegatan 19, SE-22362 Lund, Sweden; 4 Institute for Palliative Care, Lund University and Region Skåne, Scheelevägen 2, Medicon Village, SE-22381 Lund, Sweden; 5 Department of Health Sciences, Faculty of Medicine, Lund University, Margaretavägen 1B, SE-22240 Lund, Sweden; 6 Department of Public Health, Erasmus MC, Dr. Molewaterplein 40, NL-3015 GD Rotterdam, The Netherlands

**Keywords:** COVID-19, relatives, palliative care, end-of-life, quality of care, symptoms

## Abstract

Background: The COVID-19 pandemic has seen many deaths, but the majority were for causes other than COVID-19. However, end-of-life care in all settings has been affected by measures limiting the spread of the virus, for patients with and without COVID-19. The Swedish coronavirus strategy was different compared to many other countries, which might have affected end-of-life care. The aim was to describe the experiences of end-of-life care for bereaved relatives in Sweden during the “first wave” and to compare the experiences for deaths due to COVID-19 with the experiences for deaths for other reasons. Methods: A random sample of addresses for 2400 people who died during March–September 2020 was retrieved from the Swedish Person Address Registry. Relatives were contacted with a questionnaire regarding their experience of end-of-life care, with a focus on communication, participation, and trust. Results: In total, 587 relatives (25% response rate) answered the questionnaire (14% COVID-19-deaths, 65% non-COVID-19-deaths, 21% uncertain). In the COVID-19 group 28% of the relatives were allowed visits without restrictions compared to 60% in the non-COVID-19 group (*p* < 0.01). Only 28% of the relatives in the COVID-19 group reported that the person received “enough care from physicians”, significantly fewer than the non-COVID group (65%, *p* < 0.01). Conclusion: Relatives’ experience of end-of-life care for persons with COVID-19 was significantly worse than relatives of persons without COVID-19, but relatives for persons without COVID-19 were also negatively affected.

## 1. Introduction

The COVID-19 pandemic has profoundly affected the entire society and, in particular, the care of seriously ill and dying persons, those with COVID-19 as well as those dying for other reasons [1,2]. Studies of natural disasters, such as pandemics, have shown difficulties in maintaining particular key principles of palliative care such as communication, symptom management and support to relatives [3].

Reports from the media, health care professionals (HCPs) and family members have uniformly testified to the challenges of delivering high quality end-of-life care during the COVID-19 pandemic [4,5] including the decreased access of physicians in nursing homes due to restrictions. Significant barriers have been high staff workloads, lack of knowledge about palliative care, visiting restrictions for relatives, and the use of protective equipment. HCPs have expressed specific concerns regarding the risk of being infected, decreased quality of care, and shortages of medication for symptom control [6,7,8]. However, data on end-of-life care and its effect on relatives are still limited.

The Swedish COVID-19 strategy during the first year was less invasive than in many other countries, with no general lockdown. Face masks were not recommended outside health care but visits to nursing facilities were banned. The strategy focused on mitigation of the pandemic by slowing the spread of the virus, but not stopping the pandemic. In public places physical distancing was recommended, and mandatory in restaurants and during events. Daycare and schools for children up to the age of 16 stayed open and were closed for older children for several months [9]. The effect on how the Swedish strategy affected end-of-life care and bereaved relatives has not been studied.

The experiences of bereaved relatives can give crucial and unique information about how to provide the best possible end-of-life care during pandemics or other crises to come. As research in this area tends to focus on COVID-19 care only, the aim of this nation-wide observational study was to describe the experiences of relatives with respect to end-of-life care, communication, and the possibilities of being present at the bedside during the last days of life for patients dying from COVID-19 and those dying from other causes.

## 2. Materials and Methods

### 2.1. Study Design

This was a nation-wide observational study with questionnaires sent to relatives of patients who died during the COVID-19 pandemic. The study was performed in cooperation with the international CO-LIVE study group [10,11,12,13,14] and the survey was adapted from an affiliated Horizon 2020 project, the iLIVE Project (www.iliveproject.eu, accessed 1 December 2022).

### 2.2. Study Population (Recruitment and Data Collection)

A random sample was drawn from the Swedish Person Address Registry (SPAR), which keeps updated vital statistics of all inhabitants of Sweden, with the sample restricted to those who died between 15 March and 15 September 2020. In total, 2400 addresses were selected with the aim of getting in contact with the next-of-kin to those who died during the pandemic, regardless of cause of death. Questionnaires were sent in three batches during the first wave, in June, August and October 2020, as addresses from SPAR can be retrieved at the earliest one month after death. Eight hundred questionnaires were sent each time. The selected addresses corresponded to 5% of all deceased during the study period [15]. Those receiving the questionnaires, hereafter called relatives, received a letter describing the aim of the study and the questionnaire, which contained questions about end-of-life care including open-ended questions. If the questionnaire was not returned, no reminders were sent.

### 2.3. Data Collection and Categorization

#### 2.3.1. Sociodemographic Variables and Comorbidities

The relatives were asked to report information about the deceased person: age at death, gender, date of death, if the person (to the best of their knowledge) had a COVID-19 infection, and any other diseases. The COVID-19 question was asked as follows: “Was your relative ill with the new Corona virus, COVID-19?”. The question could be answered by one of five alternatives: “Yes, I am certain”, “Yes, probably”, “No, probably not”, “No, I am certain” and “I don’t know”. In the analysis, these answers were further categorized into three groups according to COVID status: (1) “Certain of COVID-19”, (2) “Certain of no COVID-19” and (3) “Uncertain of COVID-19” (which included the answers “Yes, probably”, “No, probably not” and “I don’t know”). In addition, the relatives were asked to report their own age, gender, relationship to the person, education, living arrangements and occupation/employment.

#### 2.3.2. Questions on End-of-Life Care

The questionnaire included an abbreviated version of the international Care Of the Dying Evaluation (iCODE) [16] translated to Swedish according to standard procedures; it focuses on the last two days of life and the bereavement period. The questions covered the care received from physicians and nurses, symptoms and symptom control, communication with HCPs, the emotional support from HCPs, the circumstances surrounding the person’s death, and overall impressions of care. The questions omitted from the original iCODE were about the administration of fluids and cleaning the ward, and one question about death rattle was replaced by a question on shortness of breath. To address the impact of the COVID-19 pandemic, we added self-developed questions about the impact of the pandemic on care, based on concerns that had arisen in the clinic and the media. These questions covered if and how relatives communicated with the person and the HCPs, if the relatives could visit the person during the last two days of life, and their experience with protective equipment.

### 2.4. Statistical Analysis

Characteristics of persons and relatives are captured using standard descriptive statistics (Table 1). Person characteristics are summarized for all persons and further results are presented by COVID status (3 groups). Differences between the groups are tested with χ^2^-tests (multiple proportion) or Fisher’s exact test when the expected number per cell is low. The level of missingness is in general very low (see footnote to each table) and missing observations are not imputed. Statistical significance is set at the 0.05 level. The statistical software SAS was used for all analyses (SAS Enterprise Guide version 7.1 SAS Institute, Cary, NC, USA).

## 3. Results

Of the letters sent to the 2400 addresses, 93 (4%) did not reach the intended recipient. In total, 587 relatives (25% of those invited) answered the questionnaire. Of the relatives, 68% were women and the mean age was 64 years (range 22–91). The majority (56%) were a child of the deceased person and 31% were a partner.

Of the deceased persons, 52% were women and the mean age was 82 years (range 23–108). The relatives who were certain that the deceased person was ill with COVID-19 are termed the COVID group (*n* = 81, 14%), those who were certain that the person was not ill with COVID-19 are termed the non-COVID group (*n* = 378, 65%) and those uncertain if the person had COVID-19 are called uncertain-COVID group (*n* = 126, 21%). Sex and age (≥90 years of age versus <90 years of age) were evenly distributed between the COVID-groups (*p* = 0.72 and *p* = 0.45, respectively). The most common non-COVID diseases were cancer (33%) and dementia (32%). In total, 40% died in a long-term care facility, while 28% died in hospital. (See Table 1). Only 6% died in a unit specialized in COVID-19 care. In total, 16% (*n* = 38) of those dying in long-term facilities belonged to the COVID group whereas the corresponding figure for another place of death was 12% (*n* = 43) (*p* = 0.21) (data not shown).

### 3.1. Quality of Care

Only 28% of the relatives in the COVID group reported that the person received “sufficient care from the physician,” which was significantly fewer than the non-COVID group (28% vs. 65%, *p* < 0.01) and the uncertain-COVID group (46%, *p* < 0.01). Similar results were found for care from the nurses. Additionally, trust and confidence in the physicians and nurses were significantly lower in the COVID group compared to the non-COVID group (See Table 2).

### 3.2. Symptom Burden

Regarding symptoms, more relatives in the COVID group reported that their dying relative suffered from breathlessness “all the time” (34%) compared to those in the non-COVID group (19%, *p* < 0.01). For pain and restlessness, the numbers did not differ between the COVID and non-COVID groups, but the relatives answered “I don’t know” significantly more often in the COVID group for both symptoms (See Table 2). Additionally, those not present during the last days answered, “I don’t know” more often than those present.

### 3.3. Communication

More than 80% of the relatives were informed about the person’s condition, both in the COVID- and the non-COVID group (Table 3). Significantly fewer relatives in the COVID group were involved (“very” or “somewhat”) in decision making in the last two days of life compared to the non-COVID group (43% vs. 72%, *p* < 0.01). Most of the relatives in the COVID group believed that medical treatment was limited due to the pandemic, while this was the case in only a small proportion of the non-COVID group (Table 3). Regarding time to discuss the person’s condition, significantly fewer relatives in the COVID group reported physicians and nurses having enough time, compared to the non-COVID group (Table 2). In total, 26% of the relatives in the COVID group were able to communicate in person with the patient during the last two days, compared to 57% in the non-COVID group and 41% in the uncertain-COVID group (*p* < 0.01). Similarly, it was almost twice as common for the COVID group not to have any contact at all with the person during the last two days (Table 3).

### 3.4. The Dying Phase

Relatives received information about the imminent death of their loved one in equal proportions in the COVID and non-COVID groups. Among those in the COVID group 28% were allowed visits without restrictions compared to 60% (*p* < 0.01) in the non-COVID group and 41% in the uncertain-COVID group. In total, 47% of the relatives in the COVID group reported “enough help and support” when the person died compared to 79% (*p* < 0.01) in the non-COVID group. Significantly fewer relatives in the COVID group felt close to other people the last two days before death and also after the person had died (Table 4).

## 4. Discussion

This Swedish population-based study showed that bereaved relatives of persons dying from COVID-19 during the first wave of the COVID-19 pandemic experienced significantly less involvement in decision making about end-of-life care and were less present during the last days of life compared to relatives of those dying from other causes. Most relatives of persons with COVID-19 were of the opinion that their loved ones did not receive enough medical care from the physicians and nurses and more relatives thought they did not feel trust and confidence in physicians and nurses. The persons with COVID-19 had higher levels of breathlessness, whereas other symptoms did not differ from persons with other causes of death.

Probably at least partly because of the restrictions, a minority of relatives in the COVID group were involved in decisions about care and treatment. For the relatives in the non-COVID group, it should be noted that only 70% were involved. Similar findings have been shown in the study of Mayland et al., which included persons dying from all causes during the pandemic [17]. Involving relatives in decision making requires time and knowledge about the person’s situation. During the first wave of the pandemic both lack of knowledge about the disease trajectory of COVID-19 and the fact that most relatives were not allowed to visit probably contributed to the lack of involving them in the process of decision making. A high-quality communication process with relatives has been shown to facilitate the bereavement process [18,19], and previous studies have shown that lack of communication at the end of life increases the risk of depression, anxiety, and complicated grief [20,21,22]. Fortunately, meaningful communication can be helpful even if the relatives are not present [23], and in our study 80% of the relatives received information about the person’s medical condition, both for those with and without a COVID-19 infection. Corresponding numbers have been found in previous studies during the pandemic [12,24].

Visiting restrictions influenced whether relatives were able to be present during the person’s last days of life. In our study, not only did most persons with COVID-19 die alone, but 30% of the persons without COVID-19 also had no contact at all with their relatives during the last two days. Similar to this result, a Swedish register study on end-of-life care reported that fewer than a fourth of the persons with COVID-19 during the first wave of the pandemic had relatives present when dying and that this absence was only partially compensated for by staff [24]. In a study from the UK, visits from relatives were allowed for fewer than half of the persons during the last two days [17]. This comparison is interesting as it could have been assumed that the Swedish COVID-19 strategy with no lockdown and more voluntary approach would have allowed relatives to be more present during end-of-life care [25]. These results might have been affected by the lack of protective equipment in Sweden in the beginning of the pandemic, making visits impossible. It should be remembered that the respondents in this study are the partner or a child of the person in 86% of the cases, and it is thus likely that they wanted to be present. During the years before the pandemic, the Swedish palliative care register reported that 20% (among both those with and without close relatives) were alone during their dying moment, but they may have had visits in the two days prior [26]. It seems safe to conclude that the proportion who died alone has been much higher during the pandemic, for those without COVID-19 as well, which is an important point as there is a universal wish not to die alone [27,28,29]. In many places visiting regulations during the pandemic for persons perceived to be dying were more allowing; however, it was a challenge for HCPs to recognize the dying phase in COVID-19 patients [17,30]. This difficulty likely contributed to the difference between the patients with and without COVID-19 in terms of being allowed visits in the last days of their life. Not being present during the dying phase may increase relatives’ feelings of guilt, anxiety [17] and distress [31]. The effect of visiting restrictions on the spread of COVID-19 should thus be weighed against its effect on the quality of end-of-life care and relatives’ coping with grief and loss.

An additional important finding was that only 28% of the relatives in the COVID group thought that the persons received sufficient care from physicians, compared to 65% in the non-COVID group. In addition, more than half of the relatives in the COVID group believed that medical care was limited because of the pandemic. These results might have been affected by the lower proportion of relatives given the opportunity to have in-person contact with the physicians in person among those in the COVID-group compared to the non-COVID group. Similar results have also been found in a previous study performed during the COVID-pandemic [16]. Lack of communication with HCPs and not being able to be present during end-of-life care might have affected the relatives’ experiences of the care given. A previous study revealed that HCPs found that care and treatment was indeed limited during the pandemic [11] and such limitations are most likely related to the high workloads during the pandemic [32], even though other factors such as shortage of medication [33] and a lack of knowledge early in the pandemic [34] could also have contributed. Probably as a consequence of the perceived lack of medical care, more relatives in the COVID group reported they did not have trust and confidence in physicians and nurses compared to the non-COVID group.

Regarding symptoms during end-of-life, those in the COVID group were significantly more likely to have breathlessness, which is known to be a prominent symptom of COVID-19 [35,36]. Additionally, a much higher proportion of relatives of those with COVID-19 believed that the HCPs didn’t do enough to relieve person symptoms (breathlessness, pain and restlessness). To achieve good symptom control, structured assessment and interprofessional teamwork is essential [37,38]. Clinical routines such as structured symptom assessment have been shown not to have been followed as strictly during the pandemic, which could explain this finding [39]. To be better prepared for future pandemics or other crisis, training in palliative care in hospitals and nursing homes should be improved. In addition, COVID-19 was a new and unknown disease during the first wave of the pandemic which made it difficult for the health care to manage symptoms and to communicate about prognosis with patients and relatives.

### Strengths and Limitations

Strengths of this study include the large number of participants and the use of a random sample when inviting relatives to answer the questionnaire. In addition, data were collected for persons with and without a COVID-19 infection. The questionnaire (iCODE) used in the study is a validated and previously used measure of end-of-life care, allowing the results to be compared to other studies. In addition, the changes made in the questionnaire are the same as in several other studies, thus increasing the ability to compare results [12,13,17]. The relatives’ beliefs of higher levels of breathlessness in the COVID group suggests that the reporting of COVID status was accurate. The response rate of 25% can be considered low, reducing the generalizability of the results. However, even though the participants were at a difficult point in their lives, 25% still chose to participate, a number that can be compared to the 20% response rate of healthy controls in a recent Swedish study [40]. Due to ethical reasons, we were not allowed to send any reminders. We note that the clear majority are close relatives (partners and children) of the deceased persons. It could be assumed that some of those who chose not to answer were more distant relatives who might have been less likely to be involved in the care of the person, even without pandemic restrictions. Moreover, there is no information about those who didn’t answer the questionnaires, as the only available information was the addresses to the deceased.

## 5. Conclusions

Communication, trust and being present during end-of-life in the pandemic has, according to our study, been difficult to maintain for relatives of persons with COVID-19, and also for relatives of those dying due to other causes. A major concern is that bereaved relatives believe that the care of their loved one was limited due to the pandemic, with this belief possibly affecting their bereavement and their trust in the health-care system in the long term. Efforts to minimize restrictions close to death are of greatest importance for the care of the dying during pandemics and other crises.

## Figures and Tables

**Table 1:** Characteristics of deceased persons and their relatives.

		N	%
**Deceased person’s sex**			
Female		306	52
Male		275	47
Other/Do not wish to disclose		6	1
**Deceased person’s age**			
<70		80	14
70–79		137	23
80–89		199	34
≥90		171	29
**Month of death**			
March		53	9
April		101	18
May		97	17
June		104	18
July		88	15
August		84	15
September		43	8
**Place of death**			
Home		105	19
Hospital		159	28
	ICU	16	10
Long-term care facility		231	41
Specialized palliative care		48	8
Other		26	5
**Diseases ^a^**			
Cancer		193	33
CVD/heart disease		145	25
Lung disease (COPD, etc.)		83	14
Dementia		189	32
Diabetes		84	14
Other		168	29
My relative was otherwise healthy (apart from potential COVID-19)		39	7
**COVID-19 status** (Was your relative ill with the new Corona virus, COVID-19?)			
Yes, I am certain		81	14
Yes, probably		14	2
No, probably not		87	15
No, I am certain		378	65
I do not know		25	4
**Relationship between deceased person and relative**			
The deceased person was the parent		322	56
The deceased person was the partner		179	31
The deceased person was another family member		73	13
**Relative’s sex**			
Female		398	68
Male		180	31
Other/Do not wish to disclose		9	2
**Relative’s age**			
<60		216	37
60–70		175	23
70–80		128	22
>80		68	12

^a^ Multiple answers possible, thus sums to >100%. The question was: Did you relative have any (other) diseases? Several options are possible. Missing: month of death N = 17, place of death N = 18, diseases N = 4, COVID status N = 2, relationship N = 13, education N = 10, cohabiting N = 7, occupation N = 6. None of the remining variables have any missing values.

**Table 2:** Relatives’ experience of the quality of care.

	Certain of COVID-19 N = 81		Uncertain of COVID-19 N = 126		Certain of no COVID-19 N = 378		*p*-Value
	N	*%*	N	*%*	N	*%*	
**The care from physicians was sufficient**							
Agree	22	28	56	4,6	238	65	
Neither agree nor disagree	14	18	17	14	51	14	
Disagree	18	23	18	14	32	9	
I don’t know	26	33	31	25	46	13	
							<0.01 ^b^
**The care from nurses was sufficient**							
Agree	30	38	81	67	294	80	
Neither agree nor disagree	16	20	13	11	21	6	
Disagree	10	14	8	7	25	7	
I don’t know	24	30	19	16	28	8	
							<0.01 ^a^
**I felt trust and had confidence in the physicians that cared for my relative**							
Yes, for all physicians	28	38	54	48	205	57	
Yes, for some physicians	9	12	11	10	56	16	
No, not for any of the physicians	12	16	10	9	15	4	
No physicians cared for my relative	24	33	37	33	84	23	
							<0.01 ^a^
**I felt trust and had confidence in the nurses that cared for my relative**							
Yes, for all nurses	34	44	63	54	247	68	
Yes, for some nurses	33	42	40	34	90	25	
No, not for any of the nurses	6	8	5	4	10	3	
No nurses cared for my relative	5	6	9	8	19	5	
							<0.01 ^b^
**The physicians had time to listen and discuss my relative’s condition with me**							
Agree	26	35	39	35	205	61	
Neither agree nor disagree	16	21	32	29	82	24	
Disagree	33	44	41	37	51	15	
							<0.01 ^a^
**The nurses had time to listen and discuss my relative’s condition with me**							
Agree	51	64	78	65	236	82	
Mainly agree	27	34	40	33	118	32	
Neither agree nor disagree	17	21	25	21	41	11	
Disagree	12	15	18	15	25	7	
							<0.01 ^a^
**Did your relative seem to have difficulty breathing the last two days?**							
Yes, all the time	27	34	31	25	71	19	
Yes, some of the time	15	19	33	27	111	30	
No	19	24	34	27	162	44	
I don’t know	19	24	26	21	22	6	
							<0.01 ^a^
**If there was breathing difficulty (N = 365), did the physicians and nurses do enough to relive the breathing difficulties?**							
Yes, all the time	10	16	20	23	90	45	
Yes, some of the time	10	16	21	24	47	24	
No	9	15	11	13	15	8	
I don’t know	32	53	34	40	48	24	
							<0.01 ^a^
**Did your relative seem to be in pain the last two days?**							
Yes, all the time	11	14	8	7	45	12	
Yes, some of the time	12	15	42	34	136	37	
No	22	28	42	34	150	41	
I don’t know	33	42	30	25	34	9	
							<0.01 ^a^
**If there was pain (N = 365), did the physicians and nurses do enough to relive the pain?**							
Yes, all the time	9	16	25	33	107	51	
Yes, some of the time	17	30	20	26	58	27	
No	2	4	5	7	9	4	
I don’t know	29	51	27	35	38	18	
							<0.01 ^a^
**Did your relative seem to be restless/not at peace in the last two days?**							
Yes, all the time	8	10	11	9	33	9	
Yes, some of the time	19	24	43	35	144	40	
No	23	29	32	26	146	40	
I don’t know	30	38	37	30	42	12	
							<0.01 ^a^
**If there was restlessness (N = 368), did the physicians and nurses do enough to relive the restlessness?**							
Yes, all the time	6	12	15	17	80	38	
Yes, some of the time	11	22	24	28	64	30	
No	1	2	7	8	15	7	
I don’t know	32	64	41	47	52	25	
							<0.01 ^a^

^a^ Distribution tested with multiple proportion Chi-square. ^b^ Distribution tested with multiple proportion Fischer’s exact test. Missing: The care from physicians was sufficient N = 16, The care from nurses was sufficient N = 16, I felt trust and had confidence in the physicians that cared for my relative N = 40, I felt trust and had confidence in the nurses that cared for my relative N = 24, The physicians had time to listen and discuss my relative’s condition with me N = 60, The nurses had time to listen and discuss my relative’s condition with me N = 20, Did your relative seem to have difficulty breathing the last two days? N = 15, Did the physicians and nurses do enough to relive the breathing difficulties? N = 18, Did your relative seem to be in pain the last two days? N = 19, Did the physicians and nurses do enough to relive the pain? N = 19, Did your relative seem to be restless/not at peace in the last two days? N = 17, Did the physicians and nurses do enough to relive the restlessness? N = 20.

**Table 3:** Relatives’ opinion about the quality of care and communication.

	Certain of COVID-19 N = 81		Uncertain of COVID-19 N = 126		Certain of no COVID-19 N = 378		*p*-Value
	N	*%*	N	*%*	N	*%*	
**Did you get an explanation from the health care staff regarding your relative’s condition/treatment**							
Yes	64	81	93	77	319	88	
No	15	19	28	23	43	12	
							<0.01 ^a^
**If Yes, the information was:**							
Very/rather easy to understand	48	75	81	87	300	94	
Very/rather difficult to understand	16	25	12	13	19	6	
							<0.01 ^a^
**During the last two days, how involved were you in decisions about your relative’s care and treatment?**							
Very involved	17	21	35	25	165	45	
Somewhat involved	17	21	33	27	101	28	
Not involved	46	58	58	48	102	28	
							<0.01 ^a^
**Did health care staff discuss limitations of care or treatment with you?**							
Yes, plainly	35	45	51	44	194	55	
Yes, but unclearly	19	24	15	13	28	8	
No	22	28	41	36	107	30	
I don’t know	2	3	8	7	24	7	
							<0.01 ^a^
**Do you believe that your relative’s care or treatment was limited due to the pandemic?**							
Yes	43	53	26	21	39	11	
No	24	30	69	56	278	76	
I don’t know	14	17	28	23	49	13	
							<0.01 ^a^
**Did you talk to you relative during the last two days?**							
Yes, in person	21	26	50	41	211	57	
Yes, via telephone	6	7	19	16	34	9	
Yes, via digital solutions	4	5	2	2	6	2	
No, we had no contact	50	62	50	41	117	32	
							<0.01 ^a^
**Did you have contact with the physicians?**							
Yes, in person	14	18	26	22	146	40	
Yes, via telephone/digital solutions	26	33	29	24	72	20	
No, we had no contact	25	31	48	40	85	24	
No physicians were involved in the care	15	19	18	15	58	16	
							<0.01 ^a^
**Did you have contact with the nurses?**							
Yes, in person	30	37	69	56	276	74	
Yes, via telephone/digital solutions	48	59	44	36	70	19	
No, we had no contact	3	4	10	8	25	7	
							<0.01 ^a^

^a^ Distribution tested with multiple proportion Chi-square. Missing: Did you get an explanation from the health care staff regarding your relative’s condition/treatment? N = 23, During the last two days, how involved were you in decisions about your relative’s care and treatment? N = 15, Did health care staff discuss limitations of care or treatment with you? N = 39, Do you believe that your relative’s care or treatment was limited due to the pandemic? N = 15, Did you talk to you relative during the last two days? N = 15, Did you have contact with the physicians? N = 23, Did you have contact with the nurses? N = 11.

**Table 4:** Relatives’ experience of the dying phase.

	Certain of COVID-19 N = 81		Uncertain of COVID-19 N = 126		Certain of no COVID-19 N = 378		*p*-Value
	N	*%*	N	*%*	N	*%*	
**Before your relative died, did someone tell you that death was imminent?**							
Yes	62	80	84	70	282	79	
No	16	21	36	30	74	21	
							0.101 ^a^
**Did health care staff tell you what to expect when your relative was dying (e.g., symptoms)**							
Yes	33	41	42	35	190	55	
No	47	59	77	65	158	45	
							<0.01 ^a^
**If no, would such information have been helpful?**							
Yes	19	48	28	45	50	36	
No	21	53	34	55	91	65	
							0.242 ^a^
**Were visits allowed during the last two days?**							
Yes, without restrictions	21	28	48	41	209	60	
Yes, with restrictions in number of people	16	21	31	27	75	22	
Yes, with restrictions in time of visit	6	8	2	2	8	2	
Yes, with restrictions both in people and time	3	4	2	2	5	1	
No, visits were not allowed	29	39	33	29	50	14	
							<0.01 ^b^
**I had enough help and support when my relative died**							
Agree	37	47	73	62	278	79	
Neither agree nor disagree	21	27	17	15	45	13	
Disagree	21	27	27	23	29	8	
							<0.01 ^a^
**During the last two days before my relative died I often felt ^a^**							
That I missed feeling connected to others	14	18	19	17	33	9	<0.01 ^a^
Close to other people	33	43	53	47	203	59	<0.01 ^a^
There were people I could turn to	58	74	78	67	275	78	0.07 ^a^
Others really understood me	49	62	69	62	263	75	<0.01 ^a^
**During the days after my relative died I often felt ^a^**							
That I missed feeling connected to others	18	23	20	17	42	12	<0.01 ^a^
Close to other people	37	49	55	47	218	61	<0.01 ^a^
There were people I could turn to	59	76	83	72	295	82	<0.01 ^a^
Others really understood me	50	64	76	65	260	72	0.16 ^a^

^a^ Distribution tested with multiple proportion Chi-square. ^b^ Distribution tested with multiple proportion Fischer’s exact test. Missing: information death was imminent N = 33, information on symptoms N = 40, helpful information N = 14, visits N = 4, help and support N = 39. BEFORE: connected N = 47, close to people N = 53, people to turn to N = 40, understood N = 45. AFTER connected N = 37, close to people N = 37, people to turn to N = 33, understood N = 33.

## Data Availability

The data that support the findings of this study are available from the corresponding author, [C.H.], upon reasonable request.
